# Combination of rituximab and low-dose glucocorticoids for idiopathic refractory nephrotic syndrome with MCD/FSGS: a single-center prospective cohort study

**DOI:** 10.1080/0886022X.2024.2428330

**Published:** 2024-11-15

**Authors:** Aikeda Yimamuyushan, Youqi Li, Wenwen Jiao, Jianwen Yu, Jianbo Li, Yongjun Shi, Wei Chen, Junbing He, Qinghua Liu

**Affiliations:** a Department of Nephrology, The First Affiliated Hospital, Sun Yat-sen University, Guangzhou, China; b NHC Key Laboratory of Clinical Nephrology (Sun Yat-sen University), Guangdong Provincial Key Laboratory of Nephrology, Guangzhou, China; c Department of Nephrology, Huizhou Central People’s Hospital, Huizhou, China; d Department of Nephrology, Jieyang People’s Hospital, Jieyang, China

**Keywords:** Idiopathic refractory nephrotic syndrome, rituximab, glucocorticoids, efficacy

## Abstract

**Background:**

The treatment of idiopathic refractory nephrotic syndrome (IRNS) remains a difficult problem in clinical practice. This study aims to determine the efficacy and safety of combining low-dose glucocorticoids with rituximab in IRNS treatment.

**Methods:**

This prospective, single-center cohort study enrolled 60 patients who were diagnosed with refractory IRNS with minimal change disease (MCD) or focal segmental glomerulosclerosis (FSGS) and treated at First Affiliated Hospital of Sun Yat-sen University. All patients received a treatment regimen consisting of rituximab (375 mg/m^2^/week × 4) and low-dose glucocorticoids.

**Results:**

46 complete remissions and 4 partial remissions were observed within 6 months of treatment. Within 12 months of treatment, 48 patients achieved complete remission, and 4 achieved partial remission. The complete remission rate for steroid-dependent/frequently relapsing nephrotic syndrome was significantly higher than that for steroid-resistant nephrotic syndrome (88.24% *vs.* 33.33%, *p* < .01). Following up for 12 months, 16 patients relapsed, accounting for 30.76% of the total, with a mean time to relapse of 10.97 months. Compared with baseline data, 24-hour urine protein quantification, total cholesterol and triglycerides significantly decreased, while serum albumin, globulin and IgG significantly increased at 12 months after treatment. All follow-ups were without serious adverse events. Twenty-four patients experienced infusion-related adverse reactions, which could be relieved by slowing down the infusion rate or suspending the infusion. Nine patients experienced infection-related adverse reactions; six of them were relieved with antibiotic treatment, and 3 patients were controlled by symptomatic treatment.

**Conclusions:**

Rituximab combined low-dose glucocorticoids therapy is effective and safe in idiopathic refractory nephrotic syndrome.

## Introduction

Glucocorticoids (GC) are the core therapeutic drugs for primary nephrotic syndrome, and the efficacy and sensitivity of glucocorticoids vary among different pathological types. In clinical practice, steroid-resistant nephrotic syndrome (SRNS), frequently relapsing nephrotic syndrome (FRNS), and steroid-dependent nephrotic syndrome (SDNS) are collectively referred to as refractory nephrotic syndrome (RNS). These patients often require additional immunosuppressive agents for treatment [1–3]. The binding of Rituximab (RTX, a human-mouse chimeric monoclonal antibody) to CD20 ­antigen on B-cell surfaces causes a significant decrease in peripheral blood B lymphocyte count and alleviates self-antigen-antibody immune reactions [4–6]. The latest research has found that circulating antinephrin autoantibodies are common in patients with minimal change disease or idiopathic nephrotic syndrome, and they appear to be a marker of disease activity. In patients with antinephrin-associated podocytopathy, treatment with rituximab targeting B cells can deplete antinephrin autoantibodies and induce clinical remission [7]. Currently, RTX has been shown to have good efficacy in the treatment of refractory nephrotic syndrome in children [8,9]. However, using RTX alone is difficult to achieve sustained complete remission, and sequential RTX treatment can effectively reduce the recurrence rate of idiopathic refractory nephrotic syndrome (IRNS), but it is limited by its high cost and potential infection risk [10]. Currently, there is no unified opinion on the dosage of RTX in the treatment of refractory nephrotic syndrome in adults and whether it should be combined with glucocorticoids or other immunosuppressive agents. Moreover, there is a lack of prospective studies on the treatment of refractory nephrotic syndrome with rituximab combined with low-dose glucocorticoids both domestically and internationally. This study aims to explore the efficacy and safety of rituximab combined with low-dose glucocorticoids in the treatment of RNS through a prospective study, providing a theoretical basis and practical experience for the clinical treatment of refractory nephrotic syndrome.

## Methods

### Study design and patients

Patients who were diagnosed with difficult-to-treat nephrotic syndrome and visiting the Nephrology Department of the First Affiliated Hospital of Sun Yat-sen University between January 2021 and December 2022 were enrolled in this study. Inclusion criteria was as follows: (1) age between 14 and 80 years, regardless of gender; (2) confirmed diagnosis of difficult-to-treat nephrotic syndrome with a biopsy-proven diagnosis of minimal change disease (MCD) or focal segmental glomerulosclerosis (FSGS); (3) complete clinical data available. Exclusion criteria was as follows: (1) contraindication to the use of rituximab (such as platelet count < 75 × 10^9^/L, neutrophil count < 1.5 × 10^9^/L, active hepatitis, severe infection, or drug allergy); (2) organ transplant recipients; (3) patients with concomitant tumors or rheumatologic autoimmune diseases; (4) patients with progressive renal function failure (creatinine clearance rate <30 mL/min/1.73 m^2^); (5) pregnant or lactating women; (6) hereditary kidney disease. SRNS is defined as a condition where patients do not achieve complete or partial remission after at least 4 weeks of treatment with standard doses of glucocorticoids (such as prednisone); FRNS is defined as a condition where a patient experiences more than two relapses within one year after completing initial treatment or more than one relapse within 6 months after stopping treatment; and SDNS is defined as a condition where a patient experiences at least two relapses within one year after reducing or discontinuing glucocorticoids, and each relapse requires the reintroduction of glucocorticoids treatment to be controlled [1]. This research obtained approval of the Ethics Committee of the First Affiliated Hospital of Sun Yat-sen University [approval number: 2021(102)], and was performed according to the principles of the Helsinki Declaration. All patients who were included in this research signed informed consent forms.

### Treatment regimen

The enrolled patients discontinued their original immunosuppressive agents, including fludarabine, cyclophosphamide, methotrexate, cyclosporine, and tacrolimus, retaining only the original glucocorticoids. The usage of rituximab was as follows: a dose of 375 mg/m^2^ was administered intravenously once a week for a total of four times. Thirty minutes before the medication, 5 mg of dexamethasone and 20 mg of pheniramine were given to prevent allergies. The infusion rate of RTX started at 30 mL/h and gradually increased to 90 mL/h if the patients had no allergic reactions or other discomforts. Based on the patient’s tolerance and medication history prior to treatment, an initial dose of glucocorticoids ranging from 0.25 to 0.5 mg/kg/d was given, with a preference for prednisone or methylprednisolone tablets. The relevant indicators were reassessed after one month of treatment to evaluate its effectiveness. The medication dosage was adjusted according to the relief of the patient’s condition. If patients achieve complete remission within 3 months, the dosage can be reduced by 5 mg monthly. If complete remission is not achieved within 3 months, the initial dosage can be extended to reduce by 5 mg monthly after 6 months, then gradually stop the medication based on the patient’s relief condition (Supplemental Figure 1). Angiotensin-converting enzyme inhibitors/angiotensin receptor blockers (ACEI/ARB) were given in appropriate amounts to reduce urine protein and control blood pressure based on the patient’s condition. Statins or ezetimibe were given to lower blood lipids based on baseline lipid levels. Calcium supplements and active vitamin D were given to prevent osteoporosis and other complications. If necessary, antiplatelet or anticoagulant drugs were given to prevent thrombotic complications. For some patients, prophylactic use of acyclovir or compound sulfamethoxazole was given depending on their actual situation.

### Follow-up and monitoring

After the first use of rituximab, laboratory tests including but not limited to complete blood count, serum albumin, serum creatinine, urinalysis, and 24-h urine protein quantification were performed monthly. Additionally, tests were repeated at 3, 6, 9, 12, 18, and 24 months after initial treatment, including but not limited to complete blood count, serum albumin, serum creatinine, urinalysis, 24-h urine protein quantification, B lymphocyte subset count, lipid panel, humoral immunity panel, hepatitis B virus DNA quantification, hepatitis B virus serology, cytomegalovirus IgM and IgG, and cytomegalovirus DNA quantification.

### Primary outcome

The primary outcome contains partial and complete remission. Proteinuria reduced to 0.3 g/d or UPCR less than < 0.3 g/g, stable serum creatinine and serum albumin >35 g/L set as complete remission, partially remission as decreased UPCR by 50% from baseline, and proteinuria reduced to 0.3–3.5 g/d or UPCR 0.3–3.5 g/g. A spot collection was used to determine all UPCRs.

### Secondary outcomes

Changes in total cholesterol, serum albumin, serum IgG levels, serum creatinine, proteinuria, and triglycerides between baseline and one year after the initial therapy were evaluated as secondary outcomes. UPCR values >3 g/g were defined as clinical relapse after achieving partial or complete remission. A detailed review of all relapses was conducted to distinguish clinically insignificant fluctuations in UPCR values from clinical relapses. The number of relapses, time to relapse, and subsequent treatment were recorded during follow-up.

### Adverse events

Monitoring of hospitalizations and organ- or life-threatening events is carried out in the period of follow-up. At each patient visit, the treating physician obtained details of the events, including those involving hospitals outside our organization.

### Statistical analysis

For normally distributed continuous data, mean ± standard deviation (SD) was used to express, while median (interquartile range) was used for non-normally distributed continuous data. Two-group comparisons were performed using the t-test or Mann-Whitney U test, and a paired t-test was used for before-after comparisons. Frequency or percentage (%) was used to represent categorical data, and the chi-square test was used for intergroup comparisons. Safety evaluation mainly relied on descriptive statistics, recording the types, frequency, and occurrences of adverse events. A Kaplan-Meier analysis was performed to calculate the time to complete and partial remission after the first rituximab infusion. Analysis was conducted using the statistical software packages R 3.3.2 (http://www.R-project.org, The R Foundation) and Free Statistics software version 1.8. A statistically significant difference was set as *p* < .05.

## Results

### Baseline characteristics

**Figure 1.**
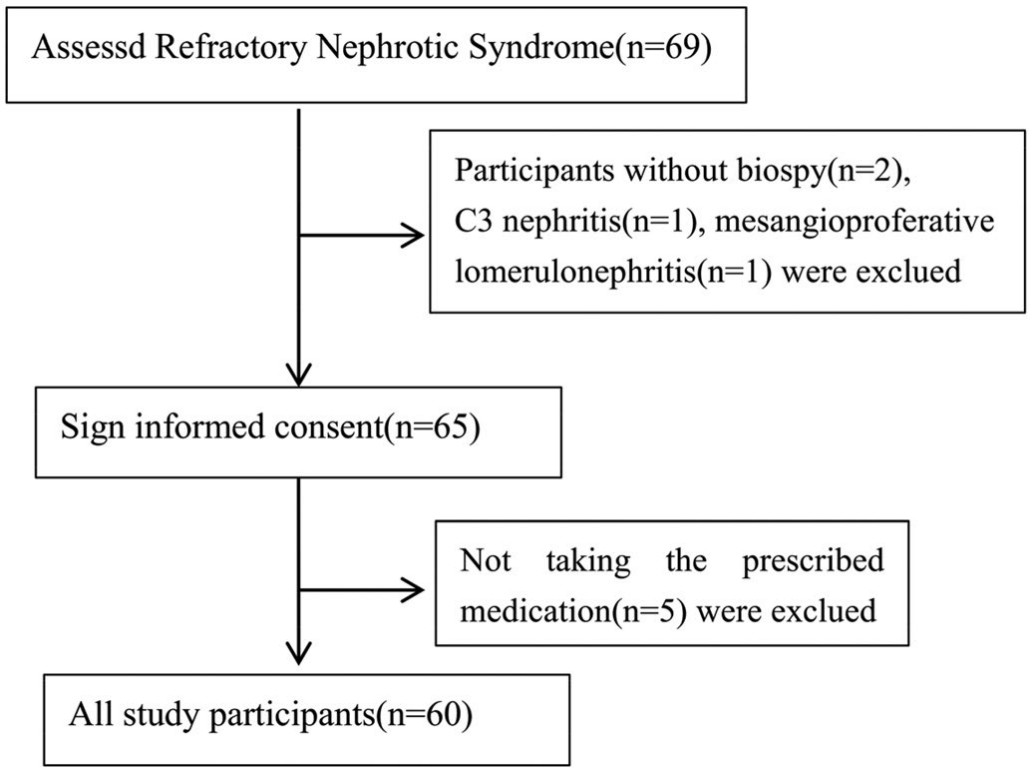
CONSORT flowchart of the study. The figure shows the CONSORT flowchart of the study.

From January 2021 and December 2022, 60 patients with refractory nephrotic syndrome diagnosed in our hospital were enrolled in this study, including 45 patients with MCD and 15 patients with FSGS. The flowchart of this study was shown in Figure 1. The baseline clinical data of the included patients was shown in Table 1. The median age of the patients was 19.5 years, including 43 males (71.7%) and 17 females (28.3%). The average body mass index (BMI) was 23.0 ± 4.5 kg/m^2^. The average duration of nephrotic syndrome history was 4.5 years, and the median follow-up time was 18.9 months. Among the 60 patients with refractory nephrotic syndrome, 9 patients (15%) had SRNS, and 51 patients (85%) had SDNS/FRNS. Of the included patients, 28 had received tacrolimus, 22 had received cyclosporine, 19 had received MMF, and 9 had received cyclophosphamide. 9 cases of SRNS, upon regimen modification, 5 cases remained without relief, whereas 4 patients experienced partial remission. For FRSDNS patients, the previous treatment was capable of inducing either complete or partial remission. Baseline data for MCD and FSGS patients showed no significant differences in gender distribution, age, BMI, baseline systolic and diastolic blood pressure, 24-h urinary protein level, blood albumin, blood cholesterol, blood creatinine, and eGFR (all *p* > .05). There was no significant difference in rituximab dosage and initial glucocorticoids use record between the two groups. Compared to the MCD group, the proportion of patients with steroid-resistant disease was significantly higher in the FSGS group (40.0% *vs.* 6.7%, *p* = .005), with a longer follow-up time (22.1 *vs.* 16.9 months, *p* = .04).

**Table 1.** Baseline characteristics of selected participants

Variables	Total (*n* = 60)	MCD (*n* = 45)	FSGS (*n* = 15)	*p* value
Age (years)	19.5 (18.0, 31.0)	21.0 (18.0, 32.0)	18.0 (16.0, 25.5)	.090
Gender [*n* (%)]				
Male	43 (71.7)	31 (68.9)	12 (80)	.151
Female	17 (28.3)	14 (31.1)	3 (20)	
BMI (kg/m^2^)	23.0 ± 4.5	23.4 ± 4.8	21.9 ± 3.5	.291
Course of diseases (months)	4.5 (1.0, 10.0)	3.0 (1.0, 10.0)	8.0 (1.5, 10.0)	.441
Follow up time (months)	18.9 (14.0, 25.9)	16.9 (13.2, 24.1)	22.1 (18.2, 30.3)	.040
RTX dose (mg)	3200.0 ± 1281.9	3164.4 ± 1237.5	3306.7 ± 1447.9	.713
RASi (ACEI/ARB) [*n* (%)]	21 (30.5)	15 (33.3)	6 (40.0)	.757
Prednisone [*n* (%)]	49 (81.7)	36 (80.0)	13 (86.7)	.714
Methylprednisolone [*n* (%)]	11 (18.3)	9 (20.0)	2 (13.3)	.714
Baseline glucocorticoid dose (mg/d)				
Prednisone	25 (15, 30)	25 (15, 30)	25 (20, 30)	.357
Methylprednisolone	16 (16, 24)	16 (16, 24)	16 (16, 24)	.436
Tacrolimus [*n* (%)]	28 (46.7)	18 (40.0)	10 (66.7)	.134
Cyclosporine [*n* (%)]	22 (36.7)	17 (37.8)	5 (33.3)	.757
MMF [*n* (%)]	19 (31.7)	14 (31.1)	5 (33.3)	1.000
Cyclophosphamide [*n* (%)]	9 (15.0)	5 (11.1)	4 (26.7)	.208
Clinical Classfication [*n* (%)]				
SDNS/FRNS	51 (85.0)	42 (93.3)	9 (60.0)	.005
SRNS	9 (15.0)	3 (6.7)	6 (40.0)	
Active nephrotic syndrome [*n* (%)]	54 (90.0)	39 (86.7)	15 (100.0)	.321
SBP (mmHg)	120.8 ± 13.0	120.8 ± 13.0	120.7 ± 13.2	.968
DBP (mmHg)	79.8 ± 11.8	79.3 ± 12.6	81.3 ± 9.1	.567
Globulin (g/L)	20.6 ± 4.3	21.3 ± 4.4	18.4 ± 2.9	.023
Albumin (g/L)	22.1 ± 8.9	23.1 ± 9.5	19.1 ± 6.2	.137
White Blood cell count (×10^9^/L)	10.1 ± 3.3	10.1 ± 3.3	10.2 ± 3.3	.881
Lymphocyte (×10^9^/L)	2.6 ± 1.1	2.7 ± 1.1	2.5 ± 1.1	.616
Red blood cell count (×10^9^/L)	4.7 ± 0.9	4.8 ± 0.9	4.4 ± 0.9	.157
Haemoglobin (g/L)	134.3 ± 24.1	135.7 ± 24.1	130.0 ± 24.5	.430
Platelet (×10^9^/L)	336.1 ± 126.1	338.2 ± 131.8	329.8 ± 110.9	.825
Cholesterol (mmol/L)	9.4 ± 4.5	9.4 ± 4.9	9.6 ± 3.5	.859
HDL-c (mmol/L)	2.1 ± 0.6	2.0 ± 0.6	2.1 ± 0.7	.638
LDL-c (mmol/L)	5.8 ± 3.1	5.8 ± 3.2	5.9 ± 2.9	.907
Proteinuria (g/24h)	6.6 (2.2, 13.2)	6.5 (1.8, 13.1)	7.5 (3.6, 13.6)	.361
Triglycerides (mmol/L)	1.9 (1.3, 2.9)	1.8 (1.0, 2.6)	2.5 (1.4, 3.5)	.100
Serum creatinine (μmol/L)	69.5 (53.0, 86.0)	70.0 (57.0, 83.0)	66.0 (49.5, 110.0)	.657
eGFR (mL/min/1.73 m^2^)	119.5 (103.2, 133.9)	119.6 (103.4, 131.7)	118.1 (79.7, 140.3)	.905
Uric acid (mmol/L)	398.0 (311.5, 461.5)	358.0 (307.8, 431.0)	462.0 (318.0, 528.0)	.069
IgA (g/L)	1.7 (1.2, 2.2)	1.9 (1.4, 2.2)	1.4 (1.1, 1.6)	.023
IgM (g/L)	1.3 (0.8, 1.7)	1.3 (0.9, 1.8)	1.3 (0.7, 1.6)	.417
IgG (g/L)	3.8 (2.1, 7.2)	3.8 (2.1, 7.5)	3.6 (2.0, 4.3)	.312
C3 (g/L)	1.0 (0.8, 1.2)	1.0 (0.9, 1.2)	1.0 (0.9, 1.1)	.680
C4 (g/L)	0.2 (0.2, 0.3)	0.2 (0.2, 0.3)	0.2 (0.2, 0.3)	.646

BMI: body mass index; RASi: Renin-Angiotensin System inhibitor; ACEI: angiotensin-converting enzyme inhibitor; ARB: angiotensin-receptor blocker; SDNS/FRNS: steroid-dependent nephrotic syndrome (SDNS) and frequently relapsing nephrotic syndrome (FRNS); SRNS: steroid-resistant nephrotic syndrome; MCD: minimal change disease; FSGS: focal segmental glomerulosclerosis; RTX: rituximab; SBP: Systolic blood pressure; DBP: Diastolic blood pressure; HDL-c: HDL cholesterol lipoprotein; LDL-c: LDL cholesterol lipoprotein; eGFR: estimated glomerular filtration rate; IgA: immune globulin A; IgG: immune globulin G.

### Primary outcome

**Figure 2.**
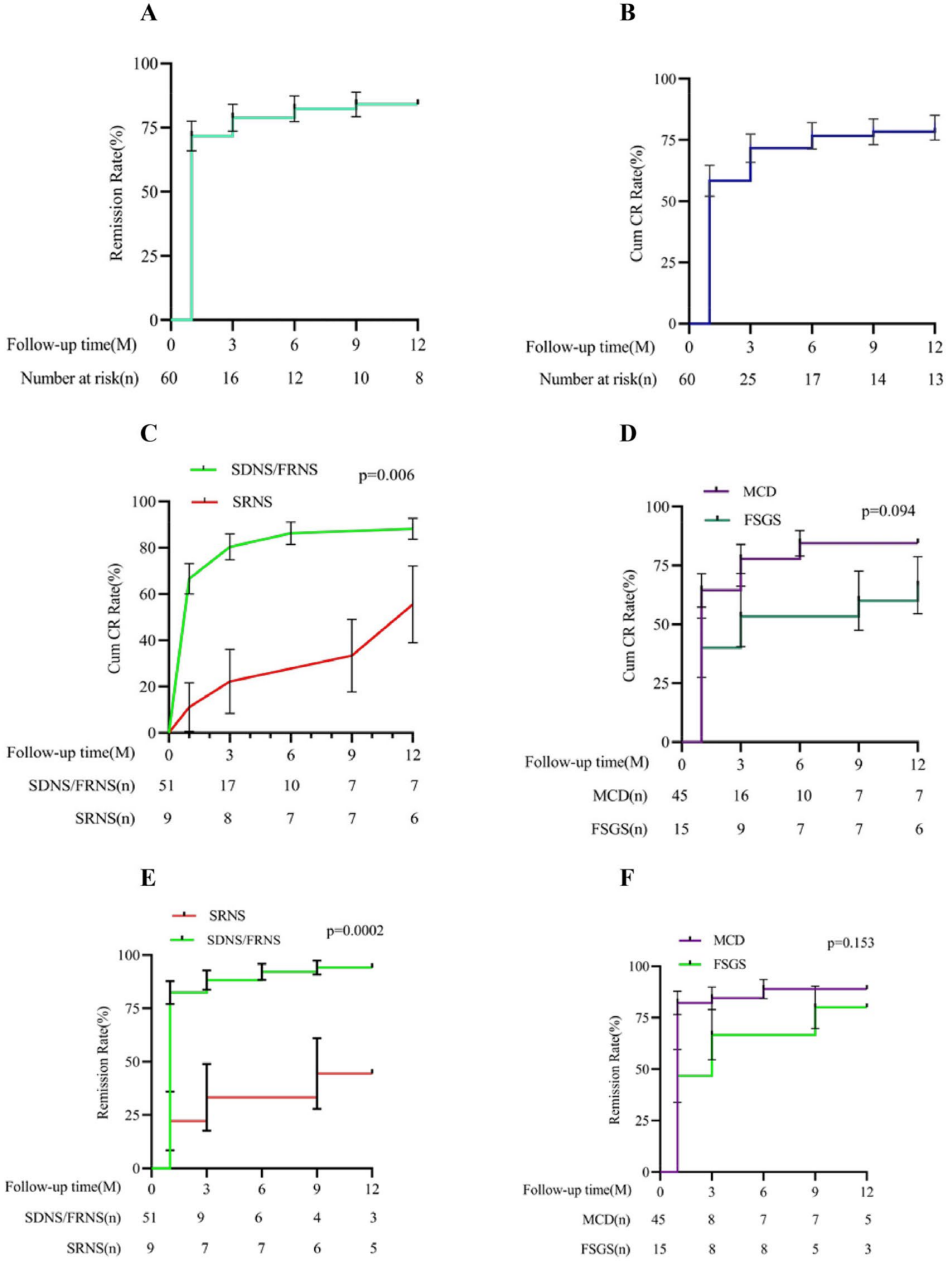
Kaplan–Meier curves for partial and complete remission. Kaplan–Meier curves for the overall group are shown for partial remission (A) and complete remission (B). Kaplan–Meier curves for the group of SNRS and SDNS/FRNS are shown for complete remission (C) and partial remission (E). Kaplan–Meier curves for the group of MCD and FSGS are shown for complete remission (D) and partial remission (F). SDNS: steroid-dependent nephrotic syndrome; FRNS: frequently relapsing nephrotic syndrome; SRNS: steroid-resistant nephrotic syndrome; MCD: minimal change disease; FSGS: focal segmental glomerulosclerosis; Cum CR: accumulative complete remission.

At one month after treatment, among the 60 included patients, 35 (58.33%) achieved complete remission, 9 achieved partial remission, and the total remission rate was 77.33%. At the 3-month follow-up, 43 patients (71.67%) achieved complete remission, 5 achieved partial remission, and the total remission rate was 80.0%. At the 6-month follow-up, 46 patients achieved complete remission, 4 achieved partial remission, and the total remission rate was 83.33%. At the 12-month follow-up, 48 patients (80%) achieved complete remission, 4 achieved partial remission, and the total remission rate was 86.67%. Subgroup analysis showed that at 1, 3, 6, and 12 months after medication, the complete remission rate and total remission rate of the MCD group were higher than those of the FSGS group. At each follow-up time point, the complete remission rate and overall effective rate of the SDNS/FRNS group were significantly higher than those of the SRNS group, and the difference was statistically significant (all *p* < .01; Figure 2).

### Secondary outcomes

#### Nephrotic syndrome parameters

**Figure 3.**
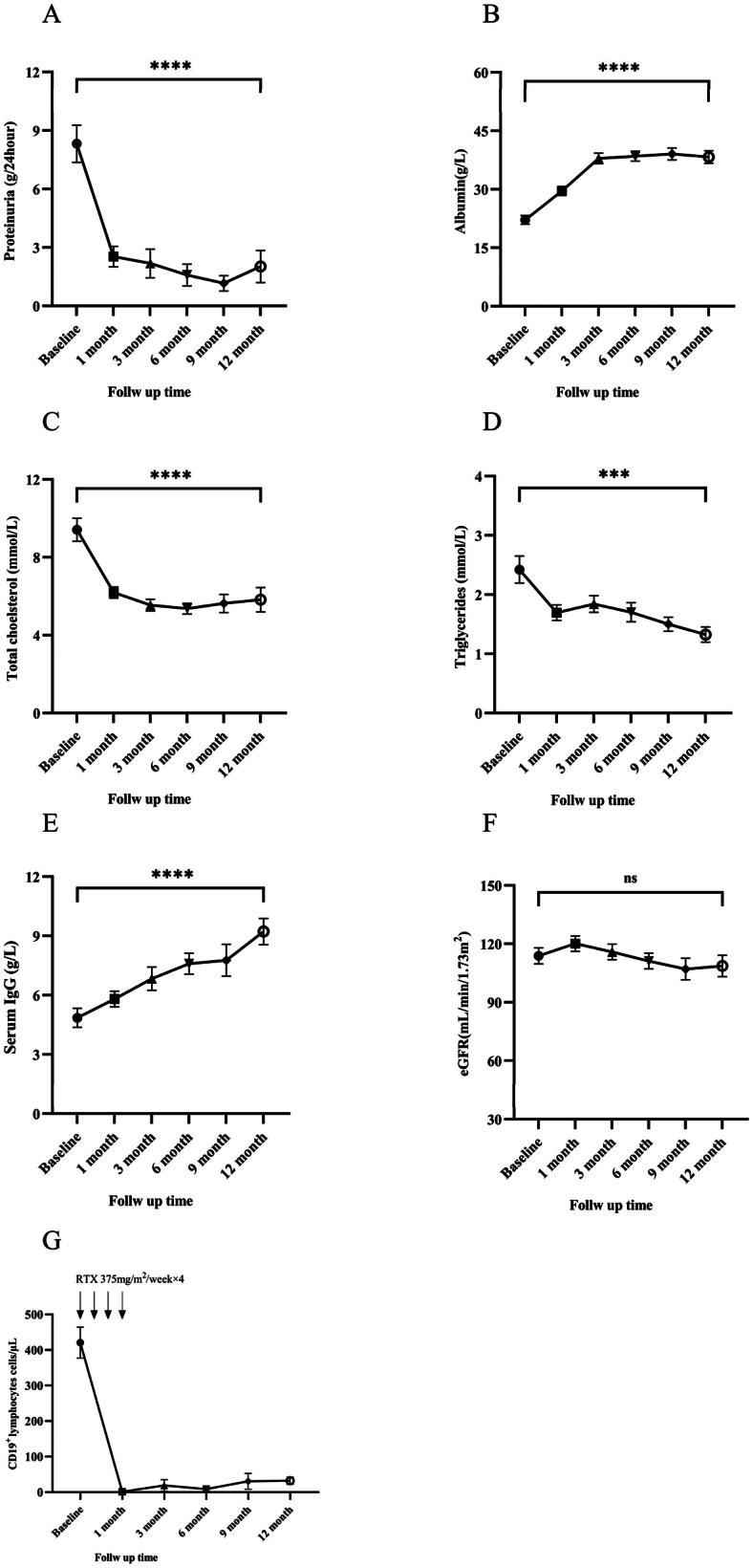
Early disease response at 12 months. Change in proteinuria (A); serum albumin (B); total cholesterol (C); triglycerides (D); serum IgG levels (E), eGFR (F) and CD19+ lymphocytes (G), from baseline to 12 months. The bars show the SEM value of the mean for each variable. Longitudinal differences from baseline to 12 months were analyzed with the Wilcoxon signed-rank test. All differences were statistically significant except for the change in eGFR (F). *p<.05, **p<.01, ***p<.001, and ****p<.0001. eGFR: estimated glomerular filtration rate; IgG: immune globulin G; RTX: rituximab.

**Figure 4.**
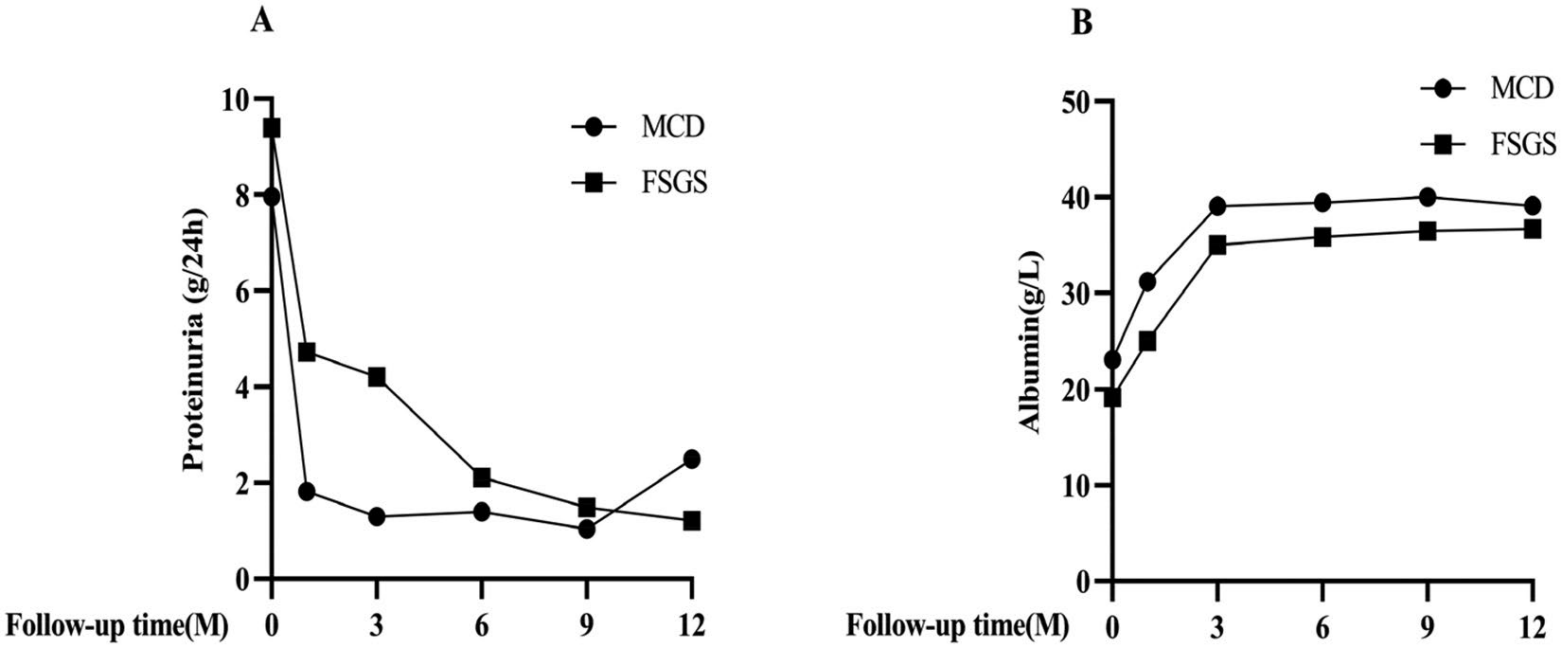
Early disease response during treatment in FSGS and MCD. (A) proteinuria; (B) serum albumin. MCD: minimal change disease; FSGS: focal segmental glomerulosclerosis.

After treatment, the 24-h urinary protein quantification, serum albumin, globulin, and serum IgG of patients gradually increased. Compared with the baseline data, there was a significant increase after 12 months of treatment, and the difference was statistically significant. Serum total cholesterol, triglycerides, and low-density lipoprotein gradually decreased. Compared with the baseline data, there was a significant decrease after 12 months of treatment, and the difference was statistically significant (Figure 3A–E). However, eGFR did not change significantly (Figure 3F). As presented in Figure 4, subgroup analysis results showed that during the follow-up period, compared with FSGS, the MCD group had a more significant increase in serum albumin and a more significant decrease in 24-h urine protein quantification.

#### B cells

CD19+ lymphocytes began to rapidly decline after the first medication, and serum CD19 + B lymphocyte levels in all patients were depleted after 4 courses of medication. Around 6 months of follow-up, an upward trend began to appear, but the average level of CD19 + B lymphocytes was still significantly lower than the baseline level at the 12-month follow-up (Figure 3G).

#### Replase

Within a period of 12 months, 52 cases attained either complete or partial remission, among which 16 cases (30.77%) experienced a relapse during the follow-up period. The average duration before relapse was 10.97 months. Notably, compared to the situation before treatment, the median number of relapses significantly dropped from 2.0 (1.3, 2.0) times to 0.0 (0.0, 1.0) times in the first six months and from 3.0 (2.0, 4.0) times to 0.5 (0.0, 1.0) times in the first year, showing a statistically significant difference (*p* < .001) (Table 2). Two of the 16 relapsed cases successfully regained remission by adjusting their glucocorticoids dosage, while 13 cases achieved remission after receiving rituximab retreatment.

**Table 2.** Comparison of pre- and post-treatment recurrence rates.

Follow up time	Pre-treatment	Post-treatment	*p* value
Six months	2.0 (1.3,2.0)	0.0 (0.0,1.0)	<.001
One year	3.0(2.0,4.0)	0.5 (0.0,1.0)	<.001

### Adverse events

Patients did not experience any serious adverse events during follow-up. 23 patients (38.33%) experienced non-serious adverse events, including adverse events during infusion and infections during follow-up (Table 3). Infusion reactions such as rash, itching, gastrointestinal reactions, tachycardia, and mild blood pressure changes could be alleviated by slowing down or temporarily suspending the infusion. Infusion reactions only occurred during the first treatment, and did not recur in subsequent treatments. During the follow-up period, 5 patients experienced respiratory tract infections, including 3 cases of upper respiratory tract infections which were alleviated after outpatient symptomatic treatment, and 2 cases of pneumonia which were alleviated after hospitalization and antibiotic treatment. Additionally, 2 cases of urinary tract infections and 2 cases of skin and mucous membrane infections were alleviated after outpatient antibiotic treatment.

**Table 3.** Adverse events during or following RTX therapy

Adverse Events	Number of Event
Adverse Events	23
Infusion reactions	10
Infection	9
Upper respiratory infection	3
Skin, subcutaneous bacterial infections	2
Urinary tract infection	2
Pneumonia (hospitalization)	2
Noninfectious diarrhea	4

## Discussion

This prospective, single-center clinical study enrolled 60 patients with IRNS to examine outcomes after a treatment regimen consisting of rituximab administered and low-dose glucocorticoids (combination therapy) over a 1-year period. Firstly, we found that the overall remission rate within 12 months of low-dose glucocorticoids and rituximab combination therapy was 86.67%, which could be achieved within a relatively short period of time. Secondly, subgroup analysis revealed that the overall remission rate of this regimen in MCD patients was higher than that of FSGS. In addition, the complete remission rate and overall effective rate of the SDNS/FRNS group were significantly higher than those in the SRNS group. Thirdly, compared to previous treatment regimens, the median number of relapses within six months and one year was significantly reduced with low-dose glucocorticoids combined with rituximab therapy. Fourthly, no sustained low IgG syndrome was found during the follow-up process, and the blood IgG level began to rise significantly three months after treatment and continued to be sustained for one year after treatment. Finally, no serious adverse events were observed during the follow-up process, indicating that this regimen has a high level of safety.

In our study, we included 60 patients with refractory nephrotic syndrome, and after 1 month of low-dose glucocorticoids and RTX combination therapy, 35 patients (58.33%) achieved complete remission. After 3 months of treatment, 43 patients (71.67%) achieved complete remission, which was significantly higher than the reported 3-month complete remission rate of 36.4% in patients with refractory nephrotic syndrome treated with glucocorticoids and tacrolimus [11]. Gulati et al. found that the 6-month complete remission rate of FK506 for refractory nephrotic syndrome was 52.4%, while the 6-month complete remission rate of low-dose RTX combined therapy was 76.67% [12]. Kazumoto et al. found that the use of rituximab in FRNS/SDNS followed by mycophenolate mofetil (MMF) could fully prevent treatment failure, with good tolerance, but the preventive effect against recurrence disappears after discontinuing MMF [13]. Our study found that the number of recurrences within half a year and one year after remission with low-dose glucocorticoids combined with RTX treatment has decreased. In summary, compared with other immunosuppressants, RTX therapy for refractory nephrotic syndrome can achieve remission more quickly. This is an important advantage of RTX, which can greatly reduce the dosage of oral glucocorticoids and other immunosuppressants and effectively reduce potential risks.

Currently, there are few studies on RTX therapy for adult steroid-resistant nephrotic syndrome in China and abroad. Our study included a total of 9 patients with steroid-resistant nephrotic syndrome, 6 of whom had FSGS. The results showed that the complete remission rate of SRNS patients treated with rituximab combined with low-dose glucocorticoids within 12 months was 33.33%, and the overall remission rate was 44.44%. This is comparable to the complete remission rate reported by Ito et al. [14] and Basu et al. [15] for the treatment of pediatric SRNS with rituximab, but significantly lower than the 70%–80% complete remission rate reported by Kamei K [16] and Bagga A [17]. In an international multicenter cohort study [18], the efficacy of rituximab in children with steroid-resistant nephrotic syndrome (SRNS) was investigated. The results showed that the 12-month remission rate was 35.1% in SRNS patients resistant to calcineurin inhibitors (CNI), while in children who received CNI treatment in the 6 months prior to rituximab administration, the 12-month remission rate was 54%. Furthermore, the study found that CR following rituximab was associated with favorable renal outcomes. The variability of response rates to RTX therapy among different studies may be due to the diversity of study designs and methodologies used by different investigators. A number of conflicting reports may be explained by factors such as the heterogeneity of SRNS, variability in the numbers and doses of RTX used, timing of RTX administration, and patients with initial or late resistance to medications. There are studies reporting that the combination of RTX and MMF is more effective than CYC in treating SRNS in children, which may be related to RTX blocking purine synthesis and enhancing the inhibitory effect of MMF on B-cell proliferation [19–21]. Therefore, for the treatment of adult SRNS patients, it may be considered to use MMF in combination with RTX.

RTX is a human-mouse chimeric monoclonal antibody that targets the CD20 antigen on the surface of normal and malignant B lymphocytes. Studies have shown that RTX primarily binds to the CD20 antigen on the surface of pre- and mature B cells, leading to B lymphocyte depletion. Our study observed that B lymphocytes began to decrease after the first intravenous administration of RTX. Although literature reports [22] that rituximab can be released into the urine during active nephrotic syndrome, leading to insufficient rituximab dosage and poor treatment outcomes, our research found that B lymphocytes were essentially depleted after the fourth administration, followed by rapid clinical remission. This is also the reason for the rapid onset of RTX’s effectiveness. The mechanism of RTX therapy for refractory MCD and FSGS is currently unclear, and some studies have observed a correlation between B cell reconstitution and disease relapse. However, our study did not provide enough data to support the dependence of relapse of refractory nephrotic syndrome on the recovery of B cell counts due to the small sample size and short follow-up time. Additionally, recent studies have found that the remission induced by rituximab in patients with antineprin antibody-associated nephrotic syndrome may be a result of autoantibody depletion [7]. Therefore, we will continue to expand our sample size, extend the follow-up time and measure the levels of nephrin in circulation to elucidate the mechanism of relapse of refractory nephrotic syndrome.

Although some researchers have found that sustained hypogammaglobulinemia and decreased IgG levels occur after RTX treatment [10,23,24], we did not observe persistently low levels of IgG and globulin in our study. On the contrary, the levels gradually increased after 3 months of treatment and were significantly higher than the baseline levels. This indicates that hypoalbuminemia and low IgG levels in patients with active nephrotic syndrome are largely manifestations of disease activity. Furthermore, no serious adverse reactions were observed in all patients in our study. Mild infusion reactions and allergic reactions were common, and timely symptomatic treatment effectively controlled them. The most common complication was infection, mainly respiratory tract and skin soft tissue infections, which were effectively controlled with symptomatic treatment in outpatient and antibiotic treatment during hospitalization, and did not significantly affect the treatment of the underlying disease.

This study has some limitations. Firstly, the sample size was small, and the data came from a single center without a control group. Secondly, the patients were only followed up to 12 months, which did not allow for a comprehensive assessment of long-term prognosis and replase rate. Therefore, in the future study, we intend to expand the sample size, foster collaborations with various centers, institute a control group, and extend the duration of follow-up to 24 months or beyond. Additionally, we plan to assess the levels of circulating nephrin to furnish a theoretical foundation for the mechanisms and clinical management of refractory nephrotic syndrome.

## Conclusion

This study provided evidence that low-dose glucocorticoids combined with rituximab therapy had a higher overall remission rate and safety in patients with difficult-to-treat nephrotic syndrome with SDNS/FRNS, and achieved rapid remission and reduced the risk of recurrence. It could effectively reduce the potential risks of oral glucocorticoids and other immunosuppressants.

## Supplementary Material

Supplemental Figure 1_R2.doc

## Data Availability

The authors declare that all data supporting the findings of this study are available within the paper. The results presented in this paper have not been published previously in whole or part, except in abstract format.
