# Supplementary figures and images for: Combination of rituximab and low-dose glucocorticoids for idiopathic refractory nephrotic syndrome with MCD/FSGS: a single-center prospective cohort study
Source: Ren Fail. 2024 Nov 15;46(2):2428330. doi: 10.1080/0886022X.2024.2428330 (PMC11571727; doi:10.1080/0886022X.2024.2428330)

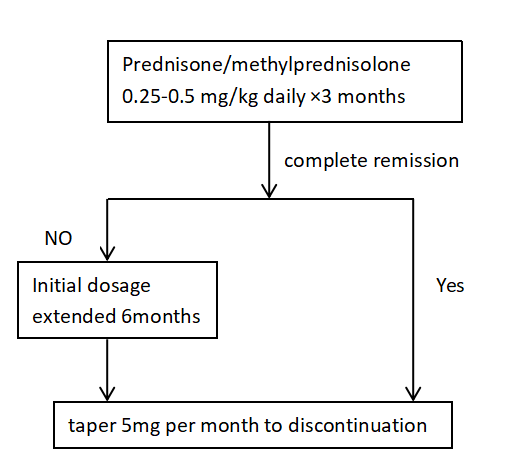


**Supplemental Figure 1**. Treatment protocol.

Supplement: Supplemental Figure 1_R2.doc [file IRNF_A_2428330_SM1284.doc]
